# Cardiac β-Adrenoceptor Expression Is Reduced in Zucker Diabetic Fatty Rats as Type-2 Diabetes Progresses

**DOI:** 10.1371/journal.pone.0127581

**Published:** 2015-05-21

**Authors:** James M. Haley, James T. Thackeray, Stephanie L. Thorn, Jean N. DaSilva

**Affiliations:** 1 Cardiac PET Centre, University of Ottawa Heart Institute, 40 Ruskin Street, Ottawa, Ontario, Canada K1Y4W7; 2 Department of Cellular and Molecular Medicine, Faculty of Medicine, University of Ottawa, Roger Guindon Hall, 451 Smyth Road, Ottawa, Ontario, Canada K1H8M5; 3 Department of Radiology, Radio-Oncology and Nuclear Medicine, University of Montreal, University of Montreal Hospital Research Centre (CRCHUM), 900 Rue Saint-Denis, Montréal, Québec, Canada H2X 0A9; 4 Department of Nuclear Medicine, Hannover Medical School, Carl Neuberg Street 1, 30625 Hannover, Germany; 5 Yale Translational Research Imaging Center, Yale University School of Medicine, New Haven, CT, 06520, United States of America; Northeast Ohio Medical University, UNITED STATES

## Abstract

**Objectives:**

Reduced cardiac β-adrenoceptor (β-AR) expression and cardiovascular dysfunction occur in models of hyperglycemia and hypoinsulinemia. Cardiac β-AR expression in type-2 diabetes models of hyperglycemia and hyperinsulinemia, remain less clear. This study investigates cardiac β-AR expression in type-2 diabetic Zucker diabetic fatty (ZDF) rats.

**Methods:**

*Ex vivo* biodistribution experiments with [^3^H]CGP12177 were performed in Zucker lean (ZL) and ZDF rats at 10 and 16 weeks of age as diabetes develops. Blood glucose, body mass, and diet consumption were measured. Western blotting of β-AR subtypes was completed in parallel. Echocardiography was performed at 10 and 16 weeks to assess systolic and diastolic function. Fasted plasma insulin, free fatty acids (FFA), leptin and fed-state insulin were also measured.

**Results:**

At 10 weeks, myocardial [^3^H]CGP12177 was normal in hyperglycemic ZDF (17±4.1mM) compared to ZL, but reduced 16-25% at 16 weeks of age as diabetes and hyperglycemia (22±2.4mM) progressed. Reduced β-AR expression not apparent at 10 weeks also developed by 16 weeks of age in ZDF brown adipose tissue. In the heart, Western blotting at 10 weeks indicated normal β_1_-AR (98±9%), reduced β_2_-AR (76±10%), and elevated β_3_-AR (108±6). At 16 weeks, β_1_-AR expression became reduced (69±16%), β_2_-AR expression decreased further (68±14%), and β_3_-AR remained elevated, similar to 10 weeks (112±9%). While HR was reduced at 10 and 16 weeks in ZDF rats, no significant changes were observed in diastolic or systolic function.

**Conclusions:**

Cardiac β-AR are reduced over 6 weeks of sustained hyperglycemia in type-2 diabetic ZDF rats. This indicates cardiac [^3^H]CGP12177 retention and β_1_- and β_2_-AR expression are inversely correlated with the progression of type-2 diabetes.

## Introduction

Type-2 diabetes mellitus (DM) and associated complications are increasing globally [1]. Type-2 DM is a risk factor for cardiovascular (CV) disease, even prior to clinical diagnosis [2]. Diabetic patients frequently have asymptomatic heart disease displaying subclinical diastolic dysfunction with left ventricular filling abnormalities and may develop systolic heart failure (HF) with reduced percent ejection fraction (%EF) [3,4]. Type-2 DM is characterized by insulin and leptin resistance, contributing to hyperglycemia and dyslipidemia, which are associated with altered sympathetic nervous system (SNS) signaling and CV dysfunction [5–8].

SNS activation increases norepinephrine (NE) production and release from postganglionic neurons into the synapse. Synaptic NE is tightly regulated, being metabolized and recaptured via the norepinephrine-reuptake transporter (NET) [9] or binding post-synaptic adrenergic receptors. NE binding to cardiac β-adrenergic receptors (β-AR) activates adenylate cyclase increasing cyclic adenosine monophosphate and calcium cycling, which elevates heart rate (HR) and contractility [10]. Chronic activation of the SNS as indicated by elevated circulating NE has been observed in DM and HF, and correlates with reduced NET expression and CV risk. Small animal studies assessing NET with [^11^C]hydroxyephedrine and positron emission tomorgraphy (PET) have identified a positive correlation between blood glucose, plasma NE and subsequent reductions in cardiac NET in hyperglycemic rats [11]. Reduced post-synaptic cardiac β-AR may also contribute to CV dysfunction in HF and DM [12–14]. The inverse correlation between cardiac β-AR density and hyperglycemia is well defined in models of insulin insufficiency like the streptozotocin (STZ) rat, but is not well characterized in models of type-2 DM [13–15]. Zucker diabetic fatty (ZDF) rats were selectively bred from the Zucker obese strain to exhibit hyperglycemia and have a knock out for the gene encoding the leptin receptor contributing to hyperleptinemia and increased fat mass. ZDF rats show early insulin resistance and hyperinsulinemia and progressive hyperglycemia as pancreatic β-cell failure impairs insulin secretion, similar to human type-2 DM [16,17]

4-(3-tert-Butylamino-2-Hydroxypropoxy)-Benzimidazol-2-One (CPG12177) is a non-selective β-AR antagonist [15,18]. Due to its hydrophilicity, CGP12177 binds to active receptors on the cell surface [19]. [^11^C]CGP12177 PET studies in humans have shown a decrease in cardiac β-AR *in vivo* in HF [12]. Reduced [^3^H]CGP12177 binding has been observed in the hearts of hyperglycemic STZ rats and corresponded with a reduction in β_1_-AR Western blotting [14,15]. Echocardiographic studies indicate diastolic dysfunction develops as normal SNS signaling deteriorates in the hearts of STZ rats [11]. Adverse echocardiographic features have also been observed in ZDF rats [20], but their association with changes in cardiac SNS signaling is not clear. Changes in cardiac β-AR expression have not been well studied in type-2 diabetic models, such as the ZDF rat. We hypothesize that the progression of type-2 DM in ZDF rats will correspond with a parallel decrease in cardiac β-AR density as measured by *ex vivo* biodistribution studies using [^3^H]CGP12177 and Western blotting. This downregulation is expected to develop with the deterioration of left ventricular function measured by echocardiography.

## Materials and Methods

[^3^H]CGP12177 (specific activity 41.6 Ci/mmol) was purchased from Perkin Elmer Health Sciences (Toronto, ON, Canada). Antibodies against rat β_1_-AR (Ab3546) were purchased from AbCam (Cambridge, MA, USA). Antibodies against β_2_-AR (SC-570), β_3_-AR (SC-1473), and GAPDH (SC-32233) were purchased from Santa Cruz Biotechnologies (Santa Cruz, CA, USA). Secondary horseradish peroxidase conjugated IgG antibodies goat anti-rabbit (SC-2004), donkey anti-goat (SC-2020), and donkey anti-mouse (SC-2314) were also purchased from Santa Cruz Biotechnology.

### Animals

Animal experiments were conducted in accordance with the recommendations of the Canadian Council on Animal Care and with the approval of the Animal Care Committee of the University of Ottawa. Male Zucker lean (ZL) (n = 16) and ZDF rats (n = 22) were obtained from Charles River Canada (Montreal, QC) between 8 and 16 weeks of age and housed individually or in pairs and maintained on a 12h light/dark cycle with *ad libitum* access to food and water. Rats were fed a diabetogenic diet (Purina 5008) consisting of 27% protein, 17% fat, and 56% carbohydrate by kcal for the duration for the study.

Fed state blood glucose and body mass were monitored over the duration of the study. Diet consumptions was measured and calculated at the terminal endpoints of 10 and 16 weeks of age. Rats from each group were excluded from the [^3^H]CGP12177 biodistribution studies providing non-tritiated samples for Western blotting and fed-state measurements. A trunk blood sample was collected during terminal biodistribution from fasted animals. Echocardiography was performed at 10 and 16 weeks to assess systolic and diastolic function.

### *Ex vivo* biodistribution

*Ex vivo* biodistribution experiments were performed as described elsewhere [15]. Briefly, restrained rats were injected via the tail vein with 8μCi (0.192 nmol) of [^3^H]CGP12177 in 200μL of saline and decapitated 30 min post-tracer injection. Trunk blood was collected and used to isolate plasma. Hearts, interscapular brown adipose tissue (BAT), and quadricep skeletal muscle were rapidly excised. Hearts were dissected into left and right atria, left and right ventricular free walls, and intraventricular septum. Samples (100mg) were processed for liquid scintillation counting as described elsewhere [15]. Briefly isopropanol and H_2_O_2_ were added to solubilized tissue in quaternary ammonium hydroxide (GE Healthcare, Montreal, QC, Canada) followed by 10ml liquid scintillation fluid (GE Healthcare, Montreal, QC, Canada) with glacial acetic acid (99^+^%) then counted using a Packard Tri-Carb liquid scintillation analyzer model 2100TR (Meriden, CT, USA). Total uptake was expressed as [(cpm recovered/g tissue) / (cpm injected/g body mass)].

### Plasma markers

Plasma measures were performed in trunk plasma from fasted and/or fed animals. Insulin was measured in fed and fasted animals using the Rat High-Range Insulin ELISA kit (ALPCO Biotechnologies, Salem, NH, USA) [21]. Free fatty acids (FFA) were measured in fasted animals using a colorimetric quantification kit (Biovision Research Products, Mountain View, CA, USA) [22]. Leptin was measured in fasted animals via radioimmunoassay (EMD Millipore, Billerica, MA, USA) [23].

### HOMA-IR

Insulin resistance was estimated using the homeostatic model assessment of insulin resistance (HOMA-IR), as defined by the equation: HOMA-IR = [(fasting glucose (mmol/L) x fasting insulin (μIU/ml)] /22.5 [24].

### Western blotting for β-AR subtypes

To provide a comparison for *ex-vivo* biodistribution experiments, and to observed changes in β-AR subtype expression, Western blotting for cardiac β_1_-AR, β2-AR and β3-AR was performed at 10 and 16 weeks on whole heart homogenates. Hearts were rapidly removed, frozen in liquid nitrogen, hand-powdered and total protein lysate was extracted. Protein determination was performed by bicinchoninic acid assay. Protein was separated on 8% sodium dodecyl sulfate-polyacrylamide reducing gels and transfered to an Immobilin-P polyvinylidene fluoride membrane (Millipore, Belirica, MA, USA) [15]. Membranes were incubated in primary antibody: rabbit anti-β_1_-AR [15], rabbit anti-β_2_-AR [13], goat anti-β_3_-AR [13], and mouse anti-GAPDH [15], then washed in TBST and incubated in the respective horseradish peroxidase conjugated IgG secondary antibodies: goat anti-rabbit, donkey anti-goat, or donkey anti-mouse. Proteins were visualized using enhanced chemiluminescence substrate for Western blotting (Perkin Elmer Health Sciences, Toronto, ON, Canada) and the FluorChem 9900 Imaging System (AlphaInnotech/Cell Biosciences, Santa Clara, CA). Blots were analyzed using AlphaEase FC software with protein band densities normalized to GAPDH using three or more replicates per measure [15].

### Echocardiography

Echocardiography was performed at 10 and 16 weeks of age under light anesthesia (1–2% isoflurane) using the Vevo 770 high-resolution *in vivo* micro-imaging system (VisualSonis, Toronto, ON, Canada) with the RMV 716 probe at 23.5 MHz, to observed if changes in cardiovascular function accompanied changes in β-AR expression and the progression of type-2 DM. Parasternal long axis views were recorded as sequential ECG-gated M-mode sweeps (EKV-mode) generating two dimensional cines of the left ventricle. The endocardial and epicardial areas were traced on the two-dimensional parasternal long axis cines and used to calculate left ventricular volumes at end systole and end diastole. Calculations for %EF and HR were complete using VisualSonics software. Diastolic function was assessed using pulse-wave Doppler across the mitral valve from the apical four chamber view. Transmitral early to atrial flow velocity (E/A) and mitral valve deceleration (MVD) time provided an indication of diastolic function [11,20].

### Statistical Analysis

All data are presented as mean ± SD. Statistical analyses were carried out using two-tailed unpaired Student’s t-tests. Significance was set as p<0.05.

## Results

### ZDF Rat Characteristics

**Fig 1:**
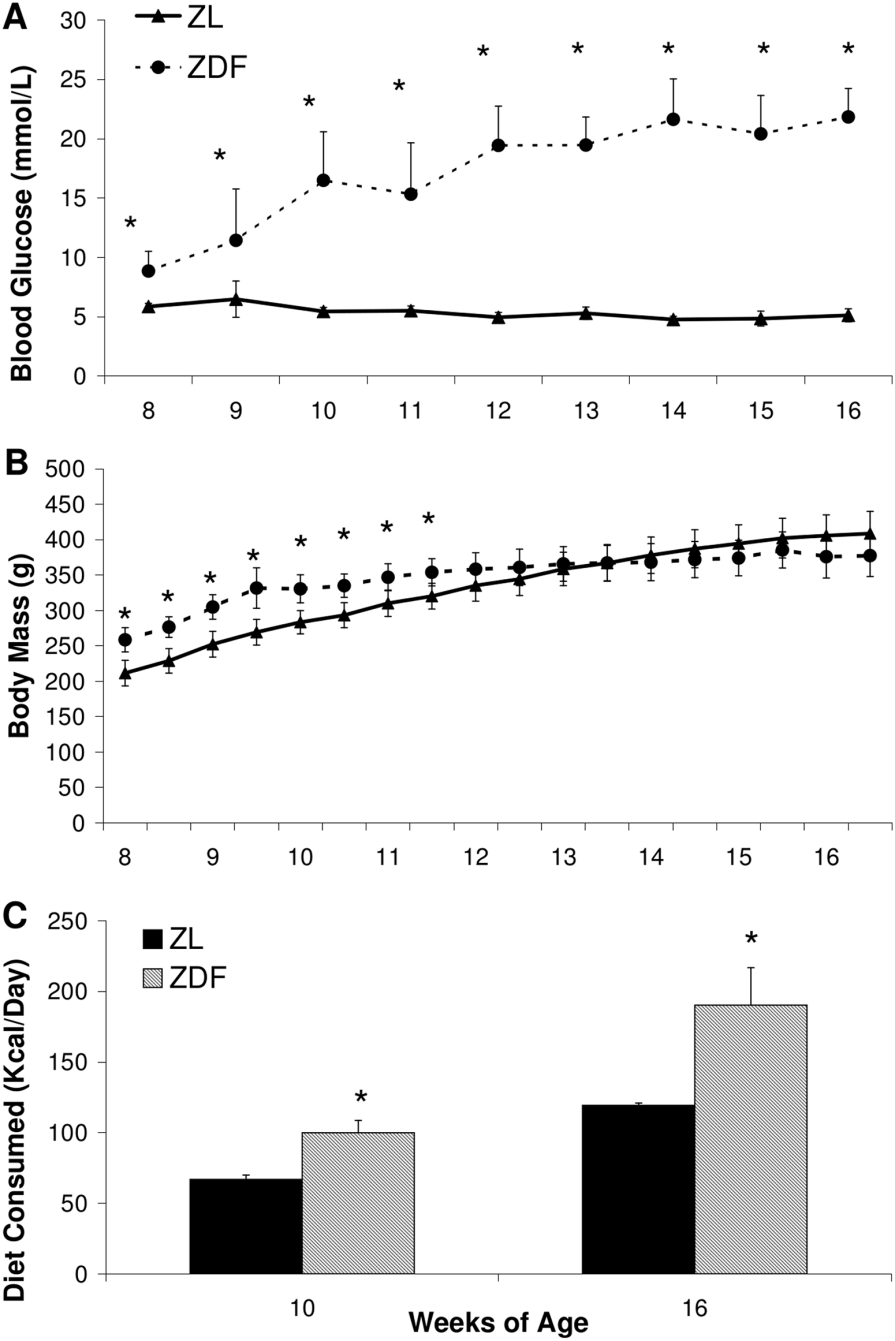
Animal model characteristics of ZDF and ZL animals. Blood glucose (A), body mass (B), and diet consumed (C). Data are mean ± SD. n = 7–10 per group. *p<0.05 vs ZL, Students t-test.

Starting from 8 weeks of age, ZDF rats exhibited moderate hyperglycemia (8.9±1.7mM) compared to ZL (5.9±0.27mM). During the course of the experiment blood glucose in ZDF gradually increased to 17±4.1 mM at 10 weeks, and began to plateau at 19±2.3 mM by 12 weeks, reaching 22±2.4 mM by 16 weeks (Fig 1A). ZDF had a significantly greater body mass than ZL controls (259±17g versus 212±18g) starting at 8 weeks of age (Fig 1B). The increase in ZDF body mass began to plateau and was not significantly greater than ZL by 12 weeks. At 16 weeks ZL rats were 409±31g and ZDF rats were 378±30g despite the qualitative appearance of greater body fat in ZDF rats. Diet consumption in kcal per day was calculated at 10 and 16 weeks in line with terminal biodistribution experiments. At 10 weeks ZDF animals consumed significantly more than ZL controls (99±8.7 versus 67±3.2 kcal/day respectively), a trend that was exaggerated by 16 weeks (190±27 kcal/day versus 120±1.4 kcal/day) (Fig 1C). HOMA-IR was significantly elevated in ZDF rats by 68% and 75% at 10 and 16 weeks of age respectively (Table 1).

**Table 1:** Plasma Insulin, FFA, Leptin, and HOMA-IR Measurements.

	Fed		Fasted							
Group	Insulin (ng/mL)		Insulin (ng/mL)		FFA (mmol/L)		Leptin (ng/mL)		HOMA-IR	
	10 weeks	16 weeks	10 weeks	16 weeks	10 weeks	16 weeks	10 weeks	16 weeks	10 weeks	16 weeks
**ZL**	1.94±0.30	3.15±0.94	1.69±0.41	1.55±0.41	0.08±0.02	0.06±0.01	2.55±1.1	5.28±0.55	11.6±3.7	10.6±2.8
**ZDF**	6.89±1.2*	2.57±0.42	3.94±1.4*	1.88±0.68*	0.34±0.12*	0.29±0.02*	30.7±2.7*	25.1±9.0*	36.7±11*	42.8±24*

FFA: free fatty acids; HOMA-IR: homeostatic model of insulin resistance; Insulin was measured in the fed and fasted states, FFA and Leptin were measured in the fasted state only. HOMA-IR was calculated from fasted glucose and insulin. Data are mean ± SD

*p<0.05 to ZL, Student's t-test; n = 3 in fed state and n = 4–7 in fasted state per group.

### Plasma Insulin, FFA, and Leptin

Relative to ZL, fasted and fed state plasma insulin were significantly higher by 57 and 72% respectively in ZDF at 10 weeks. These elevations in plasma insulin were no longer apparent at 16 weeks. Fasted plasma FFA and leptin were persistently elevated in ZDF animals at 10 and 16 weeks of age compared to ZL controls (Table 1).

### [^3^H]CGP12177 Binding

**Fig 2:**
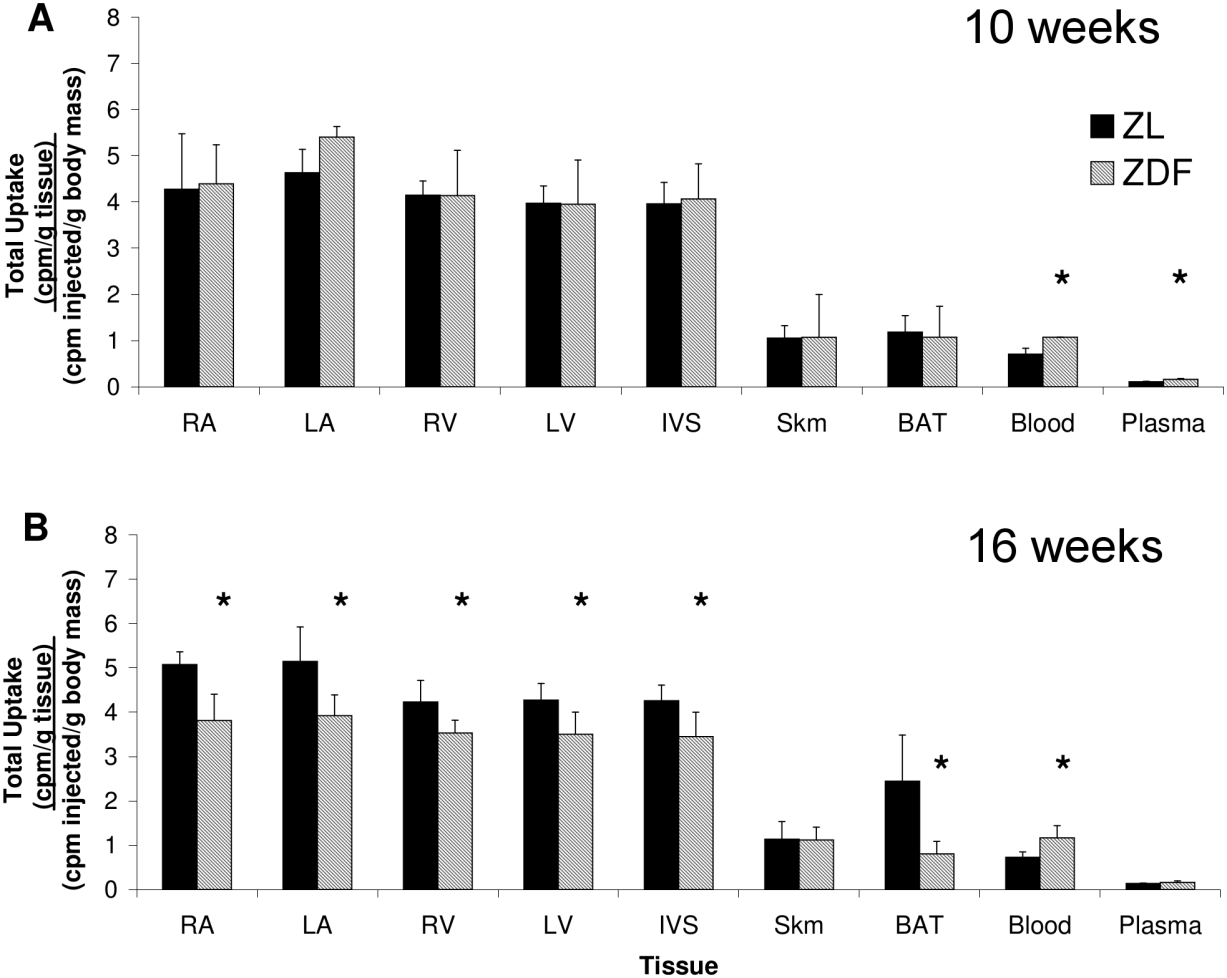
Total uptake of [^3^H]CGP12177. Total uptake [^3^H]CGP12177 in RA, LA, RV, LV, IVS, Skm, BAT, blood, and plasma of 10 (A) and 16 (B) week old ZDF and ZL. Total uptake expressed as [(cpm / g tissue) / (cpm injected / g body mass)]. RA: right atrium; LA: left atrium; RV: right ventricular free wall; LV: left ventricular free wall; IVS: intraventricular septum; Skm: quadriceps skeletal muscle; BAT: interscapular brown adipose tissue; cpm: counts per minute. Data are mean ± SD. n = 5–8 per group at each time point. *p<0.05 vs ZL, Student’s t-test.

At 10 weeks, uptake of [^3^H]CGP12177 was similar in ZL and ZDF animals across myocardial regions. No differences in [^3^H]CGP12177 binding were evident in BAT or skeletal muscle, while small but significant increases were observed in ZDF blood and plasma (Fig 2A). At 16 weeks, ZDF animals had a significant reduction in [^3^H]CGP12177 uptake in all myocardial regions of about 16–25% relative to ZL. Uptake was also 67% lower in ZDF BAT. Consistent with 10 weeks, no changes were observed in skeletal muscle uptake and small but significant increases were observed in ZDF blood (Fig 2B).

### Western blot for β-AR Subtypes

**Fig 3:**
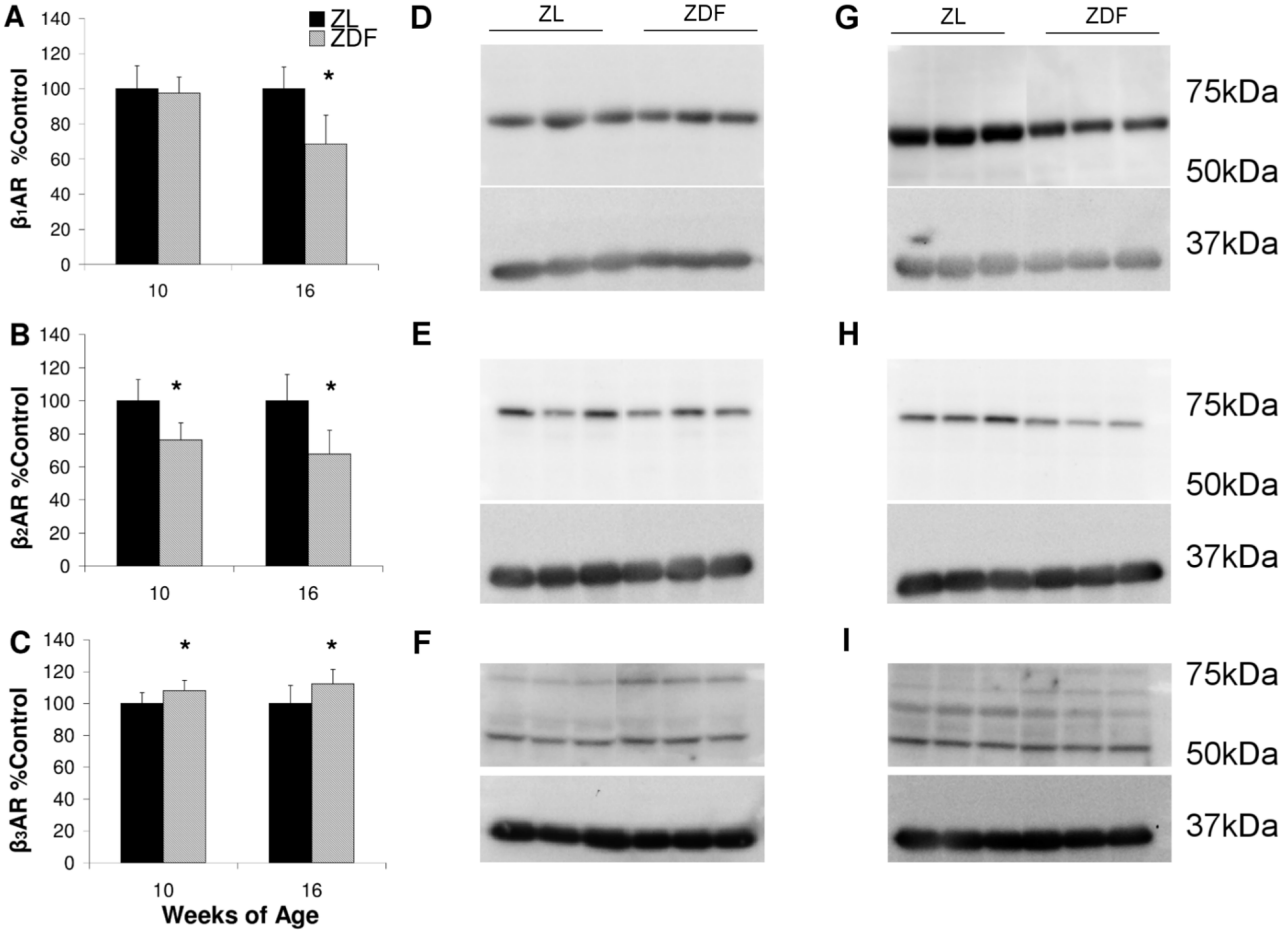
Western blot analysis of cardiac β-AR subtypes. Expression relative to ZL of cardiac β_1_-AR (A), β_2_-AR (B), and β_3_-AR (C) at 10 and 16 weeks of age. Representative 10 week blots for cardiac β_1_-AR (D), β_2_-AR (E), and β_3_-AR (E). Representative 16 week blots for cardiac β_1_-AR (F), β_2_-AR (G), and β_3_-AR (H). Relative expression compared with ZL is combined from at least 3 Western blot analyses. n = 3 per group. Data are mean ± SD. *p<0.05 vs ZL, Student’s t-test.

Western blotting at 10 weeks of age showed no difference in cardiac β_1_-AR expression between ZL and ZDF animals, but by 16 weeks β_1_-AR expression was reduced 32±16 in ZDF (Fig 3A,3D and 3G). ZDF cardiac β_2_-AR expression was 24±10% lower at 10 weeks and was decreased by 32±14% at 16 weeks relative to ZL (Fig 3B,3E and 3H). Cardiac β_3_-AR expression exhibited modest but significant elevations in ZDF animals of 8±6% at 10 weeks and 12±9% at 16 weeks of age (Fig 3C,3F and 3I).

### Echocardiography

**Fig 4:**
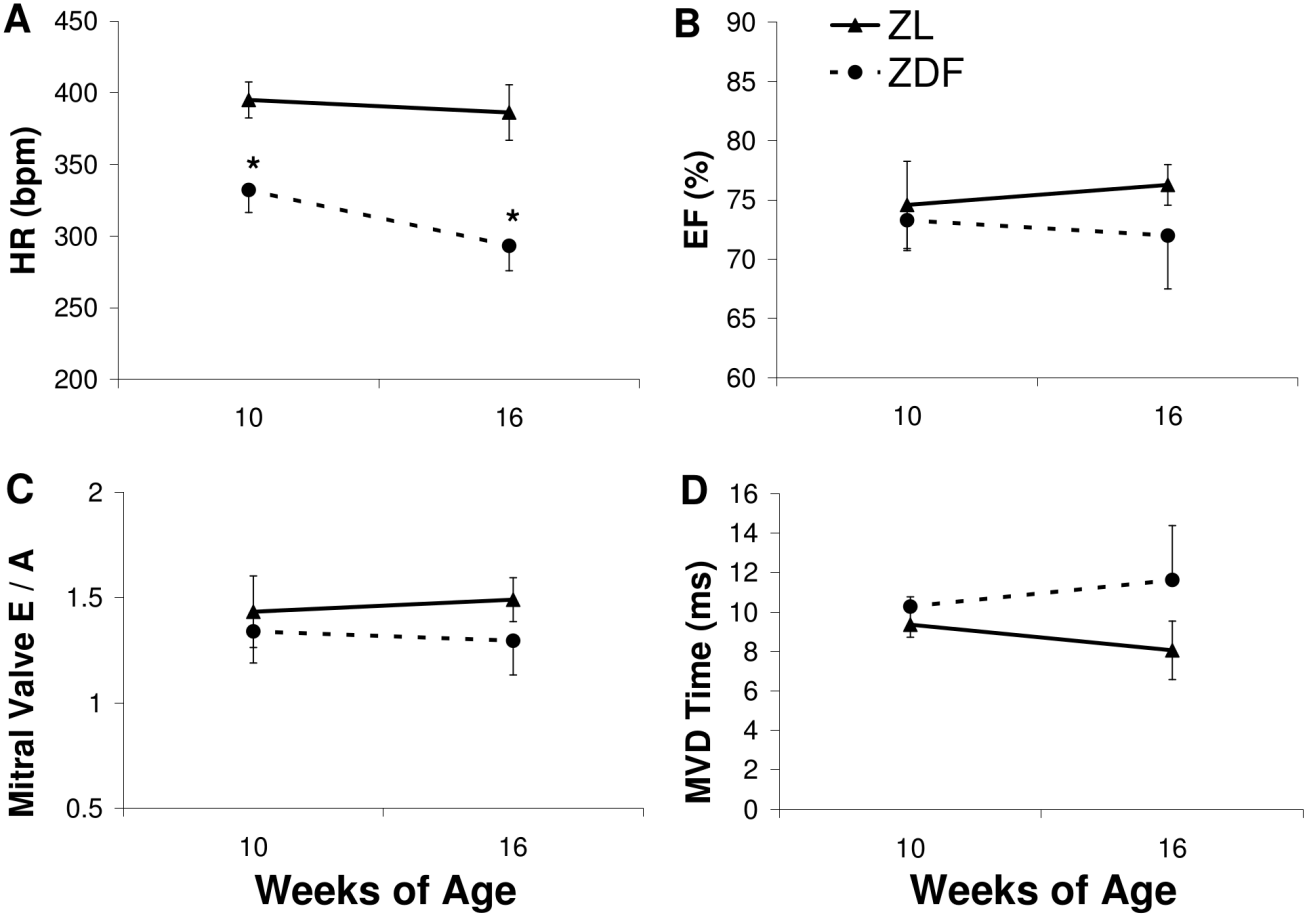
Assessment of systolic and diastolic function. Echocardiographic assessment of HR (A), %EF (B), mitral valve E/A (C), and MVD (D) in ZL and ZDF at 10 and 16 weeks of age. n = 4 per group. Data are mean ± SD. *p<0.05 vs ZL at a given time point, Student’s t-test.

ZDF animals exhibited persistent bradycardia (Fig 4A). No differences were apparent in %EF, mitral E/A, or MVD at 10 weeks. At 16 weeks of age, there were trends toward reduced %EF (p = 0.12) (Fig 4B), extended MVD time (p = 0.06) (Fig 4C), and reduced E/A (p = 0.09) (Fig 4D) in ZDF rats, but these did not reach significance.

## Discussion

Our results demonstrated that cardiac β-AR expression decreased as type-2 diabetes progressed in ZDF rats between 10 and 16 weeks of age and before systolic or diastolic function deteriorated as estimated by echocardiography. The presentation of dyslipidemia and hyperleptinemia in ZDF rats are also consistent with type-2 DM. Insulin resistance impairs myocardial energetics [25] and is associated with adverse echocardiographic features and risk of HF [26]. Reduced glucose uptake occurs in the failing myocardium, even in non-diabetics [27]. As ZDF rats age, reduced plasma insulin correlates with the progression of hyperglycemia to levels beyond those observed in younger hyperinsulemic rats [17] and appears related to β-cell apoptosis, similar to human type-2 DM [28,29]. Hyperglycemia is a risk factor for CV disease and inversely correlated with cardiac sympathetic innervation [25]. Hyperglycemia and insulin resistance in ZDF contribute to increased FFA mobilization and dyslipidemia, which correlate with CV risk in type-2 DM that can be reduced with insulin sensitizers [26]. Studies underscore the importance of hyperglycemia in altered SNS function, indicating greater plasma NE in hyperglycemic ZDF rats than in euglycemic Zucker obese rats [27] and more extensive SNS dysregulation and CV dysfunction in type-2 diabetics compared to glucose tolerant obese humans [6,7]. The relation between hyperglycemia and abnormal SNS is further supported by the observation that reducing blood glucose with insulin can attenuate cardiac autonomic neuropathy in type-1 DM patients [28] as well as reduce NE and restore cardiac NET expression in STZ rats [11]. The benefits of glycemic control on the cardiac SNS are less clear in type-2 DM [29,30]. The insulin sensitizer rosiglitazone reduces glycosilated hemoglobin in ZDF rats improving myocardial glucose utilization and reducing myocardial FFA uptake [31]. This work suggests that ZDF rats with intact insulin production may therefore be an excellent rat model for exploring the benefits of glycemic control with insulin sensitizers on the SNS and CV function in type-2 DM.

Reduced cardiac [^3^H]CGP12177 retention at 16 weeks of age but not at 10 weeks suggests β-AR expression in ZDF rats is inversely correlated with the progression type-2 DM. Reduced myocardial β-AR expression appears to be associated with reduced binding in BAT, which also exhibits high β-AR expression [32,33]. Expression of β_2_-AR on rat erythrocytes accounts for the greater activity in the blood relative to plasma in both groups [34], but it remains unclear why activity is greater in ZDF blood than ZL. Increased activity in ZDF blood may indicate changes in erythrocyte β-AR expression, however the absence of any apparent change in blood activity between 10 and 16 weeks appears to indicate this is not influencing the reduction in ZDF myocardial uptake. In the heart, β_1_- and β_2_-AR exhibit stimulatory G protein-coupling, and their downregulation is described as a compensation for hyperstimulation by NE in DM and HF [11,35,36]. β_2_-AR coupling can switch to inhibitory G protein and may thereby offer cardioprotection by limiting NE signal transduction, suggesting their reduction may contribute to cardiotoxicity [37, 38]. While reduced β_2_-AR and increased β_3_-AR expression were evident at 10 and 16 weeks, decreased [^3^H]CGP12177 binding was not evident until 16 weeks as β_1_-AR immunoblotting declined, indicating [^3^H]CGP12177 binding in the heart depended predominantly on β_1_-AR expression. The redistribution of β_2_-AR from deep transverse tubules in the healthy heart to the cell surface following myocardial infarction suggests the observed decrease in β_2_-AR may reflect a loss of receptors expressed in the transverse tubules rather than the cell surface [37, 38]. This may be why reduced β_2_-AR expression observed by Western blotting did not appear to have a large impact on [^3^H]CGP12177 binding at the cell surface. The greater dependence of [^3^H]CGP12177 binding on cardiac β_1_-AR is also supported by the greater relative expression of β_1_-AR in the healthy rat heart of about 62:30:8 compared to β_2_- and β_3_-AR [13]. *In vitro* Western blotting and reverse transcription polymerase chain reaction studies in the hearts type-1 DM STZ rats also indicate that β_1_-AR displays the greatest relative reduction [13,14]. Increased specificity of CGP12177 for β_1_-AR compared to β_2_-AR and β_3_-AR may also have an impact on this effect [14,18,32]. Elevations in cardiac β_3_-AR expression are described in DM and HF, and β_3_-AR stimulation may be cardioprotective contributing to vasodilation through endothelial nitrous oxide production [39,40]. STZ rats have normal [^3^H]CGP12177 uptake early after the induction of hyperglycemia, but binding of [^3^H]CGP12177 to cardiac β-AR becomes reduced by up to 50% after 6 to 8 weeks of sustained hyperglycemia HHHHhjhsdjfbsdlvhbasekjfvbadsrjvkbasrejfbw[14,15]. While ZDF animals present a significant and constant elevation in blood glucose similar to STZ rats, the increase in ZDF blood glucose is gradual and plateaus around 20mM compared to STZ rats that rapidly become hyperglycemic with blood glucose approaching 30mM following STZ-treatment. Consistent with less extreme hyperglycemia, ZDF rats also have intact native insulin production, while STZ rats are hypoinsulinemic [13,15]. The greater reduction in [^3^H]CGP12177 binding in STZ compared to ZDF rats indicates that cardiac β-AR are inversely proportional to the magnitude of hyperglycemia, and their reduction may be further exacerbated by the development of hypoinsulinemia in diabetic animals.

A slower HR is commonly observed in ZDF rats [27,41,42], but changes in other cardiovascular parameters such as left ventricular filling are less consistent. Millar catheter studies indicate only mild diastolic dysfunction by 44 weeks of age with a small but significant increase in the Tau constant [41], while echocardiography indicated prolonged MVD and reduced E wave velocity in 14 week old ZDF rats [20]. Similar contradictions exist in assessing systolic function in ZDF rats, with reports showing reduced %EF by 14 weeks [20,27], while others indicate normal systolic function in 44 week old ZDF [41] or even increased function in 19 week ZDF animals [42]. Given the different ages at which these studies assess CV function and the relatively similar blood glucose values, it remains unclear why these contradictions exist. Our findings appear consistent with a trend toward impaired diastolic and systolic function with a tendency toward prolonged MVD (p = 0.06), reduced E/A (p = 0.09), and reduced %EF (p = 0.12). The significant reduction in cardiac β-AR density in the absence of a significant change in cardiac function in our study indicates that CGP12177 could offer useful prognostic information prior to overt CV dysfunction in type-2 DM. CV dysfunction in diabetic patients may be difficult to detect with perfusion imaging [43], but appears to correlate well with markers of sympathetic function like iodine-123-metaiodobenzylguanidine single-photon emission computed tomography [25]. This would suggest imaging with [^11^C]CGP12177 PET may also be useful to identify patients at risk of cardiac events and help to define a therapeutic window for intervention before overt diastolic or systolic dysfunction develop.

In conclusion, binding of [^3^H]CGP12177 to cardiac β-AR is reduced between 10 and 16 weeks of age as type-2 DM progresses in ZDF rats. Western blotting indicates that while cardiac β_1_- and β_2_-AR are reduced, β_3_-AR expression is increased. ZDF rats have a slower HR as early as 10 weeks of age, with no significant changes in %EF or diastolic filling by 16 weeks. While tritium biodistribution studies underscore the potential of CGP12177 as an imaging agent, further preclinical imaging studies using a [^11^C] labeled derivative are warranted.
